# 

**Published:** 1873-02

**Authors:** 


### GEORGIA MEDICAL ASSOCIATION.

The annual meeting of the Medical Association of Georgia will take place on Wednesday, the 9th day of April, 1873, in the city of Atlanta.

J. T. JOHNSON, M.D.,
Assistant and Acting Secretary.

